# Pyrethroid and Neonicotinoid Poisoning: A Good Prognosis

**DOI:** 10.7759/cureus.47016

**Published:** 2023-10-14

**Authors:** Vikram B Vikhe, Ahsan A Faruqi, Avani Reddy, Devansh Khandol, Tejas A Kore

**Affiliations:** 1 General Medicine, Dr. D.Y. Patil Medical College, Hospital & Research Centre, Pune, IND

**Keywords:** suicide attempt, poisoning, rural india, farmer, lambda-cyhalothrin, thiamethoxam, neonicotinoid, pyrethroid, insecticide toxicity, insecticide

## Abstract

Insecticide poisoning is still one of the major means of suicide in rural India. We report a case of a 38-year-old male who had come to us with ingestion of thiamethoxam and lambda-cyhalothrin in an alcohol-intoxicated state. The prompt response and intensive care given by our center gave him a second chance to make better decisions ahead.

## Introduction

Insecticides are commonly used in India to save crop fields from being destroyed by unwanted insects and are hence readily available to a broad majority in rural India in an unregulated manner. Thiamethoxam and lambda-cyhalothrin are a newer insecticide combination of pyrethroid and neonicotinoid compounds commonly used now in India as commercial and home insecticides [1]. People are known to do unforeseen things while under the influence of alcohol. Here we discuss the management of a 38-year-old male who was brought to us by his relatives after he had ingested this insecticide in an alcohol-intoxicated state to end his life. After stabilization and counseling sessions, in the end, the patient was thankful for getting a second chance and was ready to leave his habits and have a better life ahead.

## Case presentation

A 38-year-old male was brought to our hospital by his relatives in a partially rigid posture with a gasping type of breathing. His Glasgow coma scale (GCS) on admission was 5/15 E1V2M2 with a pulse rate of 100 per minute, blood pressure of 70/50 mmHg, oxygen saturation of 60% on room air, and a blood sugar level of 66 mg/dl. Given low GCS and hypoxia, immediate endotracheal intubation was done, and mechanical ventilator support was commenced. Adequate fluid resuscitation and Inj. Noradrenaline support at 8mg in 50 ml of normal saline (NS) at 3 ml/hr was started.

History taken with the relatives revealed the patient had taken half a glass of around 100 ml of insecticide thiamethoxam and lambda-cyclothrin in an alcohol-intoxicated state in the afternoon, after which he had intractable vomiting and became unconscious. On the way to the hospital, the patient had thrown an episode of generalized tonic-clonic seizures with uprolling of the eyeballs, frothing from the mouth, and involuntary micturition in his clothes for a minute, after which his body went into a partially rigid state.

Arterial blood gas analysis of the patient upon arrival revealed metabolic acidosis with a bicarbonate deficit of 432 mmol. An injection of bicarbonate (150 mEq IV stat f/b 150 mEq IV three times a day (TDS), injection of levetiracetam (1 gm IV loading followed by 500mg IV twice a day (BD)), injection of hydrocortisone (100mg IV stat followed by 100mg IV BD), and injection of pheniramine (2 mL IV stat) were also administered. Routine blood investigations along with serum cholinesterase were sent, and the patient was kept sedated on ventilator support for one day, after which sedation was tapered (Tables 1-2).

**Table 1:** Arterial blood gas analysis upon arrival until day two ABG: arterial blood gas; pCO_2_: partial pressure of carbon dioxide; pO_2_: partial pressure of oxygen; SaO_2_: arterial oxygen saturation; sNa+: serum sodium; sK+: serum potassium; sCl-: serum chloride; sHCO3-: serum bicarbonate

ABG	Upon Arrival	Six hours later	Day two
pH (7.350–7.450)	7.19	7.34	7.42
pO_2_ (83.0–108 mmHg)	53mmHg	101 mmHg	90 mmHg
pCO_2_ (35.0–45.0 mmHg)	45mmHg	38 mmHg	40 mmHg
sHCO3 - (18–24 mmol/L)	6 mmol/L	22 mmol/L	22 mmol/L
O_2_ Saturation	62%	100%	99%
FiO_2_	Room air (0.21)	0.50	0.30
sNa^+^ (135–145 mmol/L)	138 mmol/L	140 mmol/L	142 mmol/L
sK^+^(3.5–5.5 mmol/L)	3.8 mmol/L	3.9 mmol/L	4.0 mmol/L
sCl^ -^ (96–106 mmol/L)	97 mmol/L	99 mmol/L	100 mmol/L

**Table 2:** Blood workup during the first two days of inpatient admission SGOT: serum glutamic-oxaloacetic transaminase; SGPT: serum glutamic pyruvic transaminase

Parameters (normal limit)	Day one	Day two
Hemoglobin (13.2-16.6 gm/dl)	13 gm/dl	13.2 gm/dl
Total leucocyte count (4,000-10,000 /µL)	22500 /µL	9,500 /µL
Platelets (1,50,000-4,10,000 /µL)	1,07,000 /µL	2,07,000 /µL
Serum urea (17–49 mg/dL)	28	30
Serum creatinine (0.6–1.35 mg/dL)	0.77	0.90
SGOT (8–48 IU/L)	42	43
SGPT (7–55 IU/L)	25	28
Serum bilirubin (0.2–1.2 mg/dL)	0.50	0.52
Serum cholinesterase (7000 to 19,000 U/L)	13,381	
Random blood sugar level (up to 140mg/dl)	66mg/dl	116mg/dl

Supportive management was continued; the patient was weaned from the mechanical ventilator, and a T-piece trial was given on the second day, followed by extubation on the third day. The patient was later shifted to the ward, and counseling sessions were undertaken by the psychiatrist. The patient was monitored for another three days and discharged. Follow-up has been uneventful.

## Discussion

The second most popular means of suicide in developing countries like India is poisoning [2], particularly in rural regions where poisons are readily available in the form of insecticides or fertilizers. One such biological insecticide that is most frequently employed as a commercial and home insecticide is pyrethroid. Despite being uncommon, it can be used to attempt suicide. Lambda-cyhalothrin belongs to a class of insecticides known as pyrethroids, and thiamethoxam belongs to the neonicotinoid group.

Pyrethroids cause damage to nerve and muscle cells by interacting with sodium and chloride ion channels [3]. Pyrethroids work by preventing the closure of voltage-gated sodium channels, which lowers the cell's action potential threshold, causing repetitive firing of muscle and nerve cells. In addition to reducing chloride channel current, pyrethroids can block gamma-aminobutyric acid (GABA)-gated chloride channels when present in high concentrations. Therefore, this activity causes an imbalance between synaptic inhibition and cellular excitability, which can cause a variety of neurological manifestations [4]. The lipophilic nature of pyrethroids allows them to cross the blood-brain barrier, causing neurologic effects [5]. In mild cases, symptoms include nausea, vomiting, headaches, and fine tremors. However, in severe toxicity, gross tremors, tachycardia, and hypotension are seen.

Neonicotinoids act as postsynaptic acetylcholine receptor agonists, which are neurotransmitters of the parasympathetic nervous system. Their permanent connections to these receptors initially activate, then quickly block the Na+/K+ channels and prevent the neurological influx from being transmitted. Clinical manifestations include drowsiness, gastroesophageal erosions, hemorrhagic gastritis, leukocytosis, hypoxia, and convulsions [6].

The management of both groups of insecticides is mainly symptomatic and supportive, as there is no specific antidote. Pyrethroid and neonicotinoid toxicity can be misdiagnosed because they resemble organophosphate poisoning. It is important to differentiate between these two, as pyrethroid and neonicotinoid compounds do not inhibit plasma cholinesterase, and giving atropine to these patients can cause atropine toxicity [7].

## Conclusions

With the availability of newer insecticides on the market, physicians need to be updated about the different classes of insecticides and their different clinical presentations to avoid misdiagnosis. The history of the compound ingestion is to be obtained from family members or eyewitnesses whenever possible. This case report describes acute thiamethoxam and lambda-cyhalothrin toxicity and how it can be presented with neurological manifestations, respiratory distress, and hemodynamic instability. As seen in this case, treatment is primarily supportive and has a good prognosis.

## References

[1] Zambrano ND, Arteaga W, Velasquez J, Chirinos DT (2021). Side effects of lambda cyhalothrin and thiamethoxam on insect pests and natural enemies associated with cotton. Sarhad J Agric.

[2] Kanchan T, Menon A, Menezes RG (2009). Methods of choice in completed suicides: gender differences and review of literature. J Forensic Sci.

[3] Kumar R, Selvi K (2014). Pyrethroid poisoning: an uncommon death. J Forensic Med Sci Law.

[4] Akelma H, Kilic ET, Salik F, Bicak EA, Yektas A (2019). Pyrethroid intoxication: a rare case report and literature review. Niger J Clin Pract.

[5] Panwar M, Usha G, Kumath M (2013). Status epilepticus: an association with pyrethroid poisoning. Indian J Crit Care Med.

[6] Huang NC, Lin SL, Chou CH, Hung YM, Chung HM, Huang ST (2006). Fatal ventricular fibrillation in a patient with acute imidacloprid poisoning. Am J Emerg Med.

[7] Yang PY, Lin JL, Hall AH, Tsao TC, Chern MS (2002). Acute ingestion poisoning with insecticide formulations containing the pyrethroid permethrin, xylene, and surfactant: a review of 48 cases. J Toxicol Clin Toxicol.

